# A Survey on Multimedia-Based Cross-Layer Optimization in Visual Sensor Networks

**DOI:** 10.3390/s110505439

**Published:** 2011-05-18

**Authors:** Daniel G. Costa, Luiz Affonso Guedes

**Affiliations:** 1 DCA-CT-UFRN, Campus Universitário, Lagoa Nova, Universidade Federal do Rio Grande do Norte, 59072-970 Natal RN, Brazil; E-Mail: affonso@dca.ufrn.br; 2 DTEC-UEFS, Av Transnordestina, S/N, Novo Horizonte, Universidade Estadual de Feira de Santana, 44036-900 Feira de Santana BA, Brazil

**Keywords:** cross-layer optimization, visual sensor networks, wireless multimedia sensor networks, congestion control, error recovery

## Abstract

Visual sensor networks (VSNs) comprised of battery-operated electronic devices endowed with low-resolution cameras have expanded the applicability of a series of monitoring applications. Those types of sensors are interconnected by *ad hoc* error-prone wireless links, imposing stringent restrictions on available bandwidth, end-to-end delay and packet error rates. In such context, multimedia coding is required for data compression and error-resilience, also ensuring energy preservation over the path(s) toward the sink and improving the end-to-end perceptual quality of the received media. Cross-layer optimization may enhance the expected efficiency of VSNs applications, disrupting the conventional information flow of the protocol layers. When the inner characteristics of the multimedia coding techniques are exploited by cross-layer protocols and architectures, higher efficiency may be obtained in visual sensor networks. This paper surveys recent research on multimedia-based cross-layer optimization, presenting the proposed strategies and mechanisms for transmission rate adjustment, congestion control, multipath selection, energy preservation and error recovery. We note that many multimedia-based cross-layer optimization solutions have been proposed in recent years, each one bringing a wealth of contributions to visual sensor networks.

## 1. Introduction

Wireless sensor networks (WSNs) composed of tiny low-power sensors that cooperatively operate in an *ad hoc* manner have fostered a series of innovative monitoring and control applications. Harvesting scalar information from deployed sensors in a monitored field such as humidity, pressure, temperature, luminosity, seismic variations, among others, these applications have raised many challenges that have been extensively addressed by the academic community [1–3].

Nodes in WSNs are disposable electronic devices commonly equipped with a transceiver, a limited energy supply, a sensing unity and memory and processing resources, although additional modules can be found, such as a Global Positioning System (GPS) [3]. When inexpensive low-resolution CMOS cameras and/or microphones are embedded in wireless sensors, multimedia and scalar data can be retrieved from the environment, allowing a new range of applications. The resulting Wireless Multimedia Sensor Networks (WMSNs) enhance our understanding of the physical world, improving applications like surveillance, disaster monitoring, wildlife observation, automated assistance for elderly and disabled people, traffic avoidance, industrial process control and localization services, just to cite a few [4–6]. In such contexts, when multimedia data is collected exclusively by video-based sensors, the resulting networks are referred to as Visual Sensor Networks [7].

In sensor networks comprised of visual sensors, video and/or images are collected from the environment in a different way from traditional WSNs. Most cameras installed at source nodes are not ominidirectional, resulting in a directional sensing capability usually referred as the cameras’ Field of View (FoV) [8]. In fact, the FoV is a sector-like visible region emanating from the camera, defining a direction of viewing (the camera’s pose). Viewing angle, lens quality and zoom capabilities, as well as the type of the camera used in source nodes (fixed or PTZ [8]), are major factors that influence the resulting FoV and the way visual data are retrieved from the environment.

The visual data sensed by source nodes have to be digitalized and transmitted to the sink over the sensor network. The energy and processing constraints of the sensor nodes, as well as the nature of the wireless links that interconnect them, restrict the attainable bandwidth of the communication path(s) and impose a considerable packet loss rate. Among the adopted solutions, multimedia coding techniques are used to compress the original data, reducing the required transmission rate and potentially saving energy of the source node and over the entire path(s) toward the sink. Additionally, some multimedia coding provides error resilience, which may sustain the minimum acceptable end-to-end quality of the application, even when some packets are lost while transmitted over error-prone wireless links.

The available multimedia coding techniques differ in the way they process data, directly impacting the final transmission rate, the expected end-to-end quality and the coding complexity in terms of energy consumption and required processing time and computational resources. Moreover, the nature of the target application also influences the multimedia coding, since the type of monitoring to be performed will affect the expected quality of the received media.

In visual sensor networks, it is crucial to optimize communication in order to minimize energy consumption and to maintain an acceptable quality of the application. The academic community has addressed such issues proposing multimedia-based cross-layer optimizations. In short, protocols following cross-layer design disrupt the concept of modularized layers, which reduce the overhead and optimize the jointly design of network protocols in order to meet the specific requirements of VSNs [3,6]. Following such idea, the characteristics of multimedia coding techniques have been exploited in transport, network, MAC and physical layers, influencing procedures for congestion control, error recovery, multipath selection and energy preservation.

There are many works in the literature that propose cross-layer architectures, addressing different issues of visual sensor networks. However, we classify as multimedia-based only the works that exploit the inner characteristics of the employed coding technique to achieve higher efficiency. For example, [9] is a cross-layer solution aimed at congestion mitigation based on the analysis of the memory status of sensor nodes and the current transmission trends, acting to forecast network congestion and actively adjust the transmission rate. In fact, this work presents a cross-layer architecture that according to our classification is not multimedia-based. We believe that high-efficiency VSN applications are particularly interesting in multimedia-based cross-layer optimization solutions.

Several papers can be found in the literature surveying general aspects of wireless sensor networks [1–3] and wireless multimedia sensor networks [5–7,10]. In a different way, we survey in this paper data-based cross-layer optimization solutions where the inner characteristics of the available multimedia coding techniques are exploited to achieve higher efficiency. In short, we want help to answer the following questions: (1) what are the proper multimedia coding techniques for applications, architectures, frameworks and protocols in the VSN research field? How can some multimedia coding techniques be exploited to achieve higher efficiency in terms of energy consumption, end-to-end delay and quality of received data? and (2) what are the design issues that future multimedia-based cross-layer optimization solutions should follow? In this survey, we are not concerned with the details of the algorithms and techniques for multimedia coding, which are already covered by the academic community [5,6,11].

This survey is organized as follows. Section 2 presents some fundaments of image and video coding techniques. In Section 3, the expected benefits of cross-layer design are discussed. Section 4 surveys several papers concerning multimedia-based cross-layer optimization in visual sensor networks. Future research directions are discussed in Section 5, followed by Conclusions and References.

## 2. Visual Coding Fundaments

There are many design issues that have to be properly considered when dealing with multimedia coding. If multimedia communications have to be performed over *ad hoc* sensor networks composed of nodes with energy and processing constraints, such issues become even more relevant. Some basic concepts of visual coding and their using in VSNs are discussed in this section.

The nature of packet networks such as the Internet has demanded the use of specialized multimedia coding algorithms referred as codecs (enCOder/DECoder). These algorithms aim to reduce the required transmission bandwidth of digitalized multimedia data and to allow some kind of recovery of information lost due to packet dropping. The maximum quality of the received multimedia data is obtained when the sensed data is transmitted with lossless data compression techniques and with enough redundancy to compensate packet loss. However, the resulting transmitted data will require high bandwidth, what could be prohibitive for many links. In order to achieve lower transmission rates, codecs reduce the total amount of the original data using some compression technique, demanding computational time and processing resources, but efficient data compression usually inflict some information loss. In general, information loss grows with increasing compression rate.

In visual sensor networks, some nodes endowed with a CMOS camera will transmit visual data to the sink of the network, using the *ad hoc* structure created by the deployed nodes. In such networks, the nodes are constrained in energy and processing resources and are interconnected by potentially error-prone wireless links. Such contexts create very complex environments, where the stringent requirements of multimedia communications oppose to the constrained nature of the sensor network.

In short, there are three main design objectives for multimedia coding in visual sensor networks [5,6]:

- *High compression*: Uncompressed raw multimedia data require high transmission bandwidth, which will usually not be available in the deployed sensor network. Moreover, a high bit rate also results in undesired energy consumption at the source nodes and over the currently used path(s). Thus, algorithms with efficient compression are mandatory for most VSN applications.
- *Error resilience*: The error-prone nature of *ad hoc* wireless links in wireless sensor networks can be very severe for visual monitoring. The employed coding technique can provide some error resilience, potentially improving the end-to-end attainable quality of the application.
- *Low complexity*: Multimedia coding is performed by source nodes and/or intermediate nodes which are constrained in energy and processing resources. High complex codecs may demand excessive energy consumption or be even prohibitive for a particular deployed sensor network.

We can divide multimedia data into three different categories: audio, video and still images. Audio typically represent human voice or some noise relevant to the application, with transmission rates usually lower than 64 kbps. Although audio may be important for some kind of applications on the Internet, this media is often used only to complement visual information [12] and pure wireless audio sensor networks are uncommon. In fact, most WMSN applications are concerned with the use of source sensors endowed with CMOS cameras, which are able to collect still images and videos from the environment. Still images are snapshots of the monitored target or scene, while video represent a continuous viewing created by a greater number of frames (images) transmitted and reproduced at the sink, providing a sense of motion. The required transmission rate varies according to the adopted codec, which determines the desired quality of the decoded data, the error resilience and the final resolution. Nevertheless, video is a content-rich visual media that will often require more bandwidth than still images, which has led to many works investigating image sensor networks as a less stringent communication environment for visual monitoring applications. Unless explicitly defined, video media will represent only image streams, since video-based sensors will retrieve video with no audio.

The multimedia data have to be digitalized and packetized before transmission through the network. When received at the destination, the media is converted back to its analog form, preferably as close as possible to the original media. The sources may be streaming the encoded data, turning the communication delay a major concern, or they may be transmitting data with no real-time constraints [13]. Such considerations influence the design of the communication protocols and the choosing of the proper codec for the application.

Multimedia compression can be roughly classified in lossless and lossy. In lossless compression, the digitally compressed image is identical to the original image and only low compression is obtained. On the other hand, lossy compression discards redundant information achieving higher compression. In this last case, although the compressed image is significantly different from the original image, little visible loss is perceived, if any. In WMSNs, most coding techniques are based on lossy compression.

There are a few coding techniques commonly exploited in multimedia-based cross-layer optimization. Typically, progressive and wavelet-based coding techniques are used for images, while predictive, Multiple Description Coding (MDC) and Distributed Video Coding (DVC) are encoding techniques for video. A short description of these multimedia coding techniques is presented as follows:

- *Progressive*: The source image is compressed through multiple scans with progressively increasing details. The first scan shows the image at the equivalent of a very low quality setting, and following scans gradually improve the quality. Using such coding technique, a low-quality version of the image can be exhibited very quickly, and gradual quality refinements follow. In VSN applications, a conceivable configuration is the transmission of only low quality scans, in order to save energy. The opposite of the progressive coding technique is baseline coding, which performs a single top-to-bottom scan. Typically, progressive coding is based on Discrete Cosine Transform (DCT) [11], used to reduce spatial (among neighboring pixels) and spectral (among different color planes) redundancy as much as possible.
- *Wavelet-based*: Typically, wavelet-based image compression employs the Discrete Wavelet Transform (DWT) and involves two-dimensional wavelet decomposition of the original image, giving low and high frequency subbands. Wavelet coefficients are quantized, coded and transmitted as a bit stream, achieving high compression efficiency. For VSN applications, the separate subbands are exploited to define different transmission priorities according to the relevance for the decoding process at the receiver end. Some common wavelet-based codecs are Set Partitioning in Hierarchical Trees (SPIHT), Embedded Zero-Tree (EZT) and JPEG2000 [11].
- *Predictive*: Many video codecs exploit the data statistics to reduce the transmission rate, performing intra-frame (I-frame) and inter-frame (P-frame) coding. In intra-frame coding, the redundancy within one frame is typically reduced exploiting spatial correlation, while inter-frame coding reduces the redundancy in subsequent frames exploiting both spatial and temporal correlation. MPEG-2, MPEG-4, H.263 and H.264 standards are based on predictive video coding.
- *MDC*: Multiple Description Coding fragments a single media stream into two or more substreams, which may flow in different packets through distinct paths. Each substream (description) provides acceptable low quality version of the original stream, where higher quality is obtained when all substreams are combined. The main idea is to reduce the end-to-end delay exploiting multiple paths and increase the error resilience of the communication, since packet losses will not interrupt the stream (just temporally reducing the perceived quality of the transmission). Codecs that do not generate substreams in such way are referred as single description codecs.
- *DVC*: Distributed Video Coding may significantly benefit video transmission in wireless multimedia sensor networks. In this technique, the encoders are less complex than the decoders. In other words, less energy is expected to be consumed in source nodes when using codecs based on distributed video coding, when compared with codecs based on other usual techniques. The complexity is shifted to the destination (sink), which is expected to be resource-full. The main idea of codecs based on distributed video coding is to exploit the source statistics at the decoder. For that, such coding technique takes advantage from the fact that there is a high degree of spatiotemporal information in data retrieved by visual sensors [14]. DVC is based on Slepian and Wolf’s [15] and Wyner and Ziv’s [16] works, but many variations of these original investigations can be found in the literature [17,18].

In the last few years, the research community has wondered about the proper codecs to be used in WMSN applications [3,5,6]. Initially, it has to be considered that WMSNs typically have very specialized applications, such as fire monitoring, wildlife observation and battlefield surveillance, just to cite a few. The desired quality of received data by the sink also varies according to the application, but it is reasonable to conceive that most monitoring applications will not need such good images as videoconferences, for example. The energy and processing constraints of such networks also influence the required characteristics from multimedia codecs. Nevertheless, we believe that the employed multimedia coding techniques in WMSN applications should combine high compression efficiency, low complexity and error resilience, saving energy in source node and through the entire path(s) toward the sink. The next section describes some issues of cross-layer design in wireless sensor networks. In Section 4, such issues are exploited by many multimedia-based cross-layer optimization solutions.

## 3. Cross-Layer Design

The stringent requirements of visual sensor networks have led to the design of cross-layer architectures. In order to attain high efficiency, reducing energy consumption and achieving lower communication delay, the protocols and algorithms of the MAC, network, transport and applications layers can operate in a cooperative way that disrupts the conventional data flow and the understanding of protocol layers. In such context, visual codecs play a crucial role prioritizing packets, splitting the source media in multiple streams and generating redundant information that are exploited by protocols and algorithms of different conceptual layers.

An interesting example of multimedia-based cross-layer design is just congestion mitigation by transport layer. If the classical layer organization is respected, a typical transport protocol will reduce the current transmission rate to resolve congestion issues, potentially impacting the quality of the received media by the sink (loss of video frames, higher delay, *etc*.). However, a cross-layer design may benefit from a multimedia coding technique that prioritizes the transmitted packets. In such case, the transport protocol would only reduce the transmission rate of the less-relevant packets, satisfactorily addressing congestion and potentially resulting in better end-to-end quality of the application. Many researchers advocate that cross-layer design is the best option for visual sensor networks [3,6].

Multimedia-based cross-layer optimization solutions aim to achieve at least one of the following efficiency issues in wireless sensor networks:

- *Transmission rate adjustment*: Multimedia coding techniques may prioritize the encoded data according to the relevance for the decoding process. If necessary (e.g., congestion, low bandwidth available, low energy of intermediate nodes), source nodes may avoid transmitting packets containing less relevant encoded data, decreasing the current transmission rate and saving energy throughout the path toward the sink.
- *Energy preservation*: If the current energy level of intermediate nodes is lower than a given threshold, packets carrying low-relevance encoded data may be discarded to save energy. Source nodes may also avoid transmission of low-priority packets, reducing local energy consumption and indirectly preserving energy of intermediate nodes.
- *Congestion control*: When intermediate nodes in an active path get congested, the queue algorithm can discard less relevant packets, reducing the negative impact in the perceptual quality of the received media at the sink. Also, source nodes can react to the congestion avoiding the transmission of less relevant packets.
- *Multipath routing*: The available paths in wireless sensor networks may have different characteristics in end-to-end delay and residual energy of the intermediate nodes. Prioritized packets can be routed through the “better” paths or the multipath routing facility may be used to transmit redundant packets to protect from errors during transmission.
- *Differentiated MAC transmission*: Based on the relevance of visual data after encoding, MAC protocols may provide a differentiated treatment in terms of reliability and channel access. For multimedia streaming applications, lower delays may be achieved when the link layer exploits the packets’ priorities.
- *Redundancy-based error recovery*: Multiple copies of higher priority packets may be transmitted to increase the probability of successful reception. A higher level of error-resilience is achieved when redundant packets exploit multipath routing or when intermediate nodes process/produce such packets.
- *Error recovery by correction codes*: Bit errors may corrupt data during transmission over wireless links. In such a context, a correction code like UEP (Unequal Error Protection) can be employed to protect the most relevant parts of the encoded media, where information from transport, network or MAC layers may be considered when defining the level of protection that have to be applied.
- *Retransmission-based error recovery*: Hop-by-hop retransmission can provide loss recovery with low impact in the end-to-end delay. The type and relevance of the encoded data may be used to guide the possible number of retransmissions.
- *In-network multimedia compression*: This is a cross-layer optimization in essence. When all or part of raw multimedia data is compressed by nodes other than the source, routing and transport protocols have to be employed to deliver the tasks among the nodes and coordinate the distributed compression.

Note that some of these design objectives may interrelate in some works. For example, transmission rate adjustment may directly impact the energy preservation, but reduction in energy consumption may be achieved without changes in the current transmission rate. The surveyed works in next section bring contributions in one or more of the presented design objectives.

## 4. Multimedia-Based Cross-Layer Optimization in VSNs

Modern visual sensor networks are strongly influenced by the adopted coding technique. The source transmission rate, the energy consumption over the current path(s) and the error-resilience of the communication depend on the way still images and video are encoded and decoded. What can be seen in the academic community in the last few years is a strong relation between visual coding and the communication architecture, resulting in multimedia-based cross-layer optimizations. In this paper, we classify the multimedia-based cross-layer optimization solutions in visual sensor networks in two categories:

- *Image-based*: Images are snapshots retrieved by video-based sensors, respecting the field-of-view of the embedded camera at each sensor. The image monitoring applications can be delay-unconstrained or require real-time transmission, directing impacting the adopted communication architecture. Another key aspect is that images typically require less bandwidth than video media, also consuming less energy due to lower transmission rates. The quality of the received image will also vary according to the application requirements: Grayscale low-resolution images may be suitable for some types of monitoring, while colored high-resolution images are expected for high visual definition applications or when resource-rich sensors are deployed.
- *Video-based*: Some applications directly benefit from video media to provide an enhanced understanding of the monitored target or scene. The same constraints in terms of delay and quality considered for image-based solutions are valid for video, with addition of some extremely relevant characteristics, such as the number of transmitted frames per second. The nature of video media imposes more stringent requirements for delay and jitter, also demanding more bandwidth than images. In such context, design issues as multipath routing achieve even more relevance.

Besides the type of media, we can classify the cross-layer optimization in visual sensor networks according to where the coding is performed:

- *Source processing*: When raw visual data are fully processed by the sensor which collected them, the compression is said to be performed locally at the source node. For that, the source must have sufficient processing and memory resources to properly execute the encoding algorithms. Source processing potentially reduces the end-to-end delay, but may demand considerable energy consumption from the source node.
- *In-network processing*: When source nodes do not have sufficient resources to encode a large volume of data, when the communication architecture define specialized tasks for intermediate nodes or if it is desired to save energy of source nodes, in-network processing is performed. It must be specified some protocol to share the original data among the nodes which will compress the data. In such case, the energy is saved in source nodes at the cost of additional complexity.

The next subsections survey multimedia-based cross-layer optimization considering all the previously presented design issues.

### 4.1. Image-Based Cross-Layer Optimization

When dealing with image compression, three very relevant characteristics will be typically present in the encoded images: unequal importance, error tolerance and constrained error propagation [19]. The unequal importance characteristic refers to the fact that different parts of compressed images have different perceptual and structural relevance. The error tolerance denotes that errors during transmission over the network may not prohibit the reconstruction of the original data, which may be performed even with some degradation. Finally, the constrained error propagation indicates that if some bits are corrupted, the neighboring bits are likely to become useless as well. Such characteristics are commonly considered by image-based cross-layer optimization solutions.

Visual sensor networks for image-based monitoring are very attractive since the communication requirements are less stringent than video streaming monitoring applications. Analyzing the surveyed works, we can identify different approaches for cross-layer optimization. Among the proposed solutions, wavelet-based prioritization and progressive coding seem to be very suitable for wireless image sensor networks, and new investigations concerning cross-layer design exploiting such coding techniques should still arise.

#### 4.1.1. Progressive Image Coding

Bourkerche *et al.* [20] proposed the Reliable Synchronous Transport Protocol (RSTP) for synchronization of image transmission from multiple sources, assuring the same level of quality for the received images and a fairer utilization of the available bandwidth. This is accomplished by employing the JPEG codec in progressive encoding mode, where the source images are compressed through multiple scans with progressively increasing details. Using such coding technique, a low-quality version of the image can be exhibited very quickly, and gradual quality refinements follow.

In [20], one practical effect of progressive encoding is that for low-bandwidth and error-prone environments, the images may be displayed entirely, but with low quality. This coarse-to-clear presentation mode may be more desirable than the slow top-to-bottom mode of baseline JPEG. Employing JPEG in progressive mode, RSTP can show smoothly the images with increasing precision. To perform the desired synchronization, the authors define both frame level and quality level synchronization. The frame level synchronization is concerned with the number of frames transmitted per second (fps). On the other hand, quality level synchronization adjusts parameters of the source encoding, also impacting end-to-end quality and required transmission rate.

RTSP is a TCP-based transport protocol that can synchronize the source nodes to achieve equivalent levels of quality, potentially adapting to the current state of the network (available bandwidth, packet loss rate and congestion, *etc.*). The main concept behind RSTP is the transmission management at the receiver end, where the sink controls the transmissions from the source nodes. Additionally, congestion and error control are specified in this transport protocol. The RTSP transmission management is further investigated in [21]. As a promising contribution, the authors propose a mosaic algorithm to align multiple images, achieving a larger field of view.

An interesting consideration about [20] and [21] is that both works expect that JPEG will be the dominant technique for image sensor nodes. However, the energy consumption and computational complexity of the employed codec are not investigated, nor are other possibilities considered.

Cheng and Shang [22] also investigated the JPEG codec in low-power and low-bit-rate wireless networks, exploiting progressive JPEG encoding as in [20]. In that work a coding strategy that only employs high quality encoding to parts of the image with higher relevance for the application is proposed, leaving the remaining data with huge compression (and lower quality). At the destination, all parts of the same image (potentially having some prioritized parts) are reassembled, resulting in different qualities in the same image. The amount of data encoded in one scan is equal to or less than the bandwidth of the network. Such approach is slightly different from the work in [20], which varies the quality of entire atomic images.

The image-based cross-layer optimization proposed in [22] is a priority-driven scheduling algorithm. That algorithm schedules for transmission more data from important sub-images, according to the available bandwidth. Additionally, when it is impossible to transmit the entire image within the current bandwidth and power constraints, some data pertaining to unimportant sub-images are discarded.

VSN applications can benefit for visual monitoring where priority-based JPEG encoding like the one presented in [22] is used in source nodes. For example, if applications are monitoring a moving target, such a coding strategy would potentially reduce the bandwidth requirements (and energy consumption) of transmission of less-relevant parts of the scene (as visual data of the sky and the ground), keeping the viewing of the target with high quality for the application.

Progressive image encoding is also exploited in [23]. The idea is to route more relevant packets through more reliable paths, where the path reliability is a function of the expected error rate and node failure. Instead of achieving higher transmission rates as in video-based multipath routing solutions, multiples paths toward the sink are employed in [23] to increase the probability that the transmitted images are received with quality as good as possible. For that, this work assumes that the source nodes have information about the entire network topology. Besides multipath routing, the authors propose the use of Unequal Error Protection to offer an additional level of reliability. Thus, more relevant data receives stronger protection against bit errors during transmission.

An interesting consideration presented in [23] is that higher reliability cannot be achieved just loading more reliable paths with most packets. The success of forward correction codes is based on the distribution of the packets over several paths.

#### 4.1.2. Wavelet-Based Image Coding

Wavelet-based image compression is also exploited for cross-layer optimization. The work [24] proposes an energy-efficient self-adaptive image transmission scheme, providing a trade-off between the energy consumption to transmit encoded images and the quality of the received media at the sink. The proposed scheme is based on DWT and different levels of reliable transmission. In fact, the DWT technique defines priorities for the encoded data, since the image can be decomposed into separate subbands. Typically, wavelet-based image compression involves two-dimensional wavelet decomposition of the original image, giving low and high frequency subbands. Wavelet coefficients are quantized, coded and transmitted as a bit stream. Whatever the chosen prioritization scheme in [24], full reliable transmission is only required for high-priority data, reducing the energy consumption throughout the path toward the sink. The remaining data is transmitted in a semi reliable mode.

The reliability mechanism proposed in [24] is based on the way intermediate nodes treat packets before they are relayed. When the energy resource of intermediate nodes is lower than a given threshold, low-priority packets are discarded while high-priority packets are relayed through the current path toward the sink. The expected outcome is the prolonging of the network lifetime, keeping an acceptable quality level for the received images.

In order to avoid discarding packets that have crossed many hops, resulting in an undesired waste of energy, the authors of [24] consider the energy consumption in preceding nodes. Such a consideration is further investigated in [25], which defines two general packet dropping schemes for the work in [24]. In the open-loop scheme, it is only considered the energy of the intermediate node. On the other hand, close-loop also regards the available energy in the next intermediate nodes to the sink, which could help in prediction of the dropping probability of the transmitted packets.

Lee and Jun [26] propose a computational solution to mitigate congestion by reduction on the data transmission rate with low sacrifice of the end-to-end application quality. That work defines the Adaptive Compression-based congestion control Technique (ACT) aimed at the control of congestion by a reduction in the number of transmitted packets, but keeping the received data quality. For that, ACT employs the following compression techniques: DWT, Adaptive Differential Pulse Code Modulation (ADPCM) and Run Length Coding (RLC). In fact, that work does not directly define a codec for multimedia compression, as in [19,24], but the compression algorithms used in [26] may be used for image coding, since DWT and RLC are used by some codecs of the JPEG family.

As mentioned before, the DWT technique indirectly defines priorities for the encoded data. In case of congestion, such priorities are considered by intermediate nodes. The idea is to discard packets containing less relevant data when the congested node may chose what packets must to be discarded. Additionally, ADPCM reduces the amount of transmitted data from the source using the principle of quantization, while RLC generates a smaller number of packets for low-priority data. Putting these all together, less data must be transmitted, saving energy throughout the active congested path toward the sink with low degradation in the end-to-end perceptual quality.

The work in [19] aims at adaptive reduction of energy consumption in processing and transmission of images in wireless sensor networks. Energy saving is achieved through image compression using JPEG2000 codec [27,28], according to the acceptable quality of the transmitted images. JPEG2000 codec is a wavelet-based still image compression standard that presents high compression performance and strong error resilience.

Using information about the end-to-end distortion constraints and the estimated channel condition, the proposed solution in [19] can adaptively determine the number of encoding layers to be transmitted. In other words, it is possible to adjust the source coding rate, the source level error resilience scheme and the transmitter power level. What the proposed solution does is to find the optimal number of encoding layers to be transmitted and the optimal strategies for each layer.

Although designed to be used in generic wireless sensor networks, in [19] only the case where the transmitter can directly communicate with the receiver is considered. This is the reason why the proposed solution defines adjustment of the transmitter power according to the current network condition. For many applications, sensors are expected to be randomly deployed, since it is easier and less expensive for large wireless sensor networks [8]. Moreover, it is common to expect dense deployment with a huge number of nodes. In such case, direct communication between source node and the sink is not practical, demanding excessive energy consumption and increasing the sensor costs in most of VSN applications.

The work presented in [29] exploits the overlapped area created by the field of view of source image sensors, when there are inter-sensor correlations [8]. Instead of saving energy by employing in-network compression algorithms, the authors propose that the source sensors transmit only visual data corresponding to areas with no overlapping and additionally some part of the covered overlapped area (avoiding transmission of visual information that has been already transmitted by other sources). Thus, the total transmitted data is reduced throughout the network, and the end-to-end quality of the application is preserved, since the FoV of at least two sensors overlaps in the desired communication scenario. In such case, extra processing is expected from the sink, which has to recreate the viewed images based on the received packets.

In [29] the Multi-Level Rate-Oriented Routing (MLRR) is proposed, a routing scheme where lower transmission rates should be associated with the nodes having less residual power energy, potentially prolonging the network lifetime. For that, the considered paths are node-disjoint and should respect the rate constraints of the sources.

The concept of using overlapping areas to reduce energy consumption due to lower transmission rates is very promising, but presents some drawbacks. As deployed video-based sensors may have a unique view of a target or scene, even overlapping sensors may produce different visual information [8] (for example, viewing the front and back sides of a person). On the other hand, if two or more source sensors have exactly the same view, only one has to be active at a given time. So, for some types of visual monitoring, the proposed solution may have restricted applications.

The work presented in [30] proposes a position based cross layer resource allocation approach to achieve optimal image transmission quality while assuring energy efficiency in wireless multimedia sensor networks. For that, the unequal importance among image-pixel-position information (p-data) and image-pixel-value information (v-data) is exploited, following the wavelet image compression paradigm. As p-data are more important, they receive more protection by UEP, while the relatively unimportant v-data segments are less protected, improving energy efficiency.

The loss probability reduction of p-data increases the attainable perceptual quality of the transmitted image, while the loss of v-data segments has low impact. So, the increased energy consumption for more protection on important p-data segments is compensated by the less protection on unimportant v-data segments. The level of protection on each type of coded data is subject to the energy budget on each link, which is assumed to be provided by network layer.

In that approach, in order to reduce energy consumption in multimedia processing and transmission, raw or encoded images can be processed by other nodes besides the source. The basic idea behind in-network processing is to employ (idle) intermediate nodes to share the processing load with source nodes. For that, there should be a logical structure that supports the desired in-network compression. Among the possibilities, clustering [31] is the most used approach.

**Figure 1.**
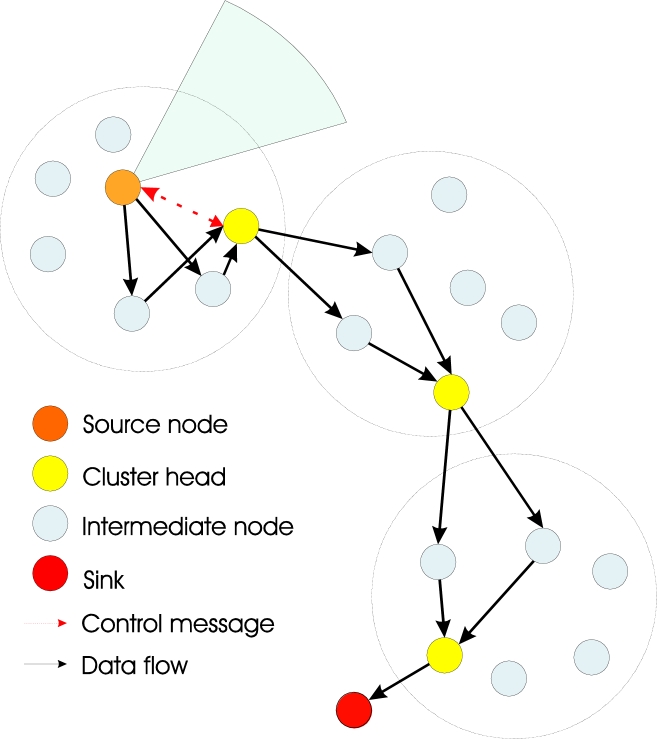
A cluster-based in-network image processing scheme.

A cluster head is selected in each cluster and maintains a membership list of the associated cluster nodes, and each cluster knows its cluster head [32]. To allow communication among clusters, each cluster head also knows the path to its neighboring clusters. In such logical structure, the cluster heads control the distribution of the data to be processed among the associated cluster nodes. The way packets flow on the network through the available clusters depends on the employed routing protocol and the correspondent routing scheme. Figure 1 shows an example of distributed cluster-based image processing where only two intermediate nodes in each cluster are employed for encoding/decoding.

Wu and Abouzeid in [33] defined a mechanism for distributed image compression in visual sensor networks. In order to support the share of the processing tasks, that work assumes a cluster-based topology for a dense deployment of nodes where only some of them are endowed with a camera (source nodes). The authors argue that individual nodes may not have sufficient computational power for source coding of all collected visual data, turning in-network image compression potentially advantageous. Even if the source nodes have sufficient computational power, in-network compression can extend the network lifetime due to energy saving just exploiting idle sensors.

The compression technique in [33] is wavelet-based and is expected to be performed by several groups of nodes along the path. The sink sends a request specifying the desired quality to the source node, which perform an initial compression (entropy coding) and send the image to its neighboring nodes for further compression, under the control of the cluster head. The in-network encoding follows the cluster structure and the required number of wavelet decomposition levels. Each cluster is responsible for a wavelet decomposition level, but it is not necessary to employ all nodes of the same cluster. The required number of wavelet decomposition levels is typically small, and some clusters may have no compression to perform or be responsible for more than one wavelet decomposition level if the sensor network is composed of few nodes.

The original images may be distributed to the nodes using two different data exchange schemes: Division by rows/columns and tiling (segmenting of original image in smaller “parts”/tiles). These schemes differ in the way the image is organized for compression.

The work in [34] proposes a distributed image compression scheme based on the JPEG2000 codec, exploiting the DWT technique and the Embedded Block Coding with Optimized Truncation (EBCOT) algorithm. As in [33], the authors argue that sensors nodes do not have sufficient computational power to compress a large volume of data, and in-network compression would be a reasonable solution to reduce energy consumption in source nodes and over the current path(s) toward the sink.

To perform in-network compression, the computational tasks must be distributed among other nodes. For that, a method to share the processing task is proposed, which employs cluster-based routing: Cluster heads distribute compression task among nodes under the same cluster. As the path from the source to the sink may pass through many clusters, different compression tasks (e.g., horizontal decomposition, vertical decomposition, *etc.*) are performed by each cluster, but some clusters may only forward the packets with no additional compression, as in [33].

The works presented in [33] and [34] provide similar results, with some slight differences. An interesting contribution of [34] is an adaptive compression scheme, where the quality of the transmitted image is subject to dynamic parameters to adapt the communication process. In such cases, different levels of transmission quality, computational complexity and energy consumption can be achieved according to the application requirements. The main contribution of both works is actually the prolonging of the lifetime of source nodes, but there is a still more promising benefit of the proposed solutions. As the amount of data is decreased along the path due to compression, the communication cost of the nodes closer to the sink is smaller than the communication cost of previous nodes of the path. In fact, if all nodes closer to the sink run out of energy, the whole network goes offline. As nodes closer to the sink receive more combined upstream traffic, energy saving in such nodes may have a deep impact in the expected network lifetime. Nevertheless, in both works the end-to-end delay of the proposed solutions is not investigated. As images are processed during transmission by many intermediate nodes, the transmission is subject to an extra delay and significant jitter. For real-time applications, the potential higher delay may render unfeasible the proposed in-network image compression.

The same in-network encoding paradigm is also employed in [35]. While the methods in [33] and [34] encode images in a distributed way, with different types of coding during transmission over the network, the work presented in [35] consider local source encoding but in-network error recovery based on redundancy and correction codecs. The similarity of these works relies on the cluster-based structure of the deployed *ad hoc* sensor network.

Wireless links in visual sensor networks are expected to be error-prone. As retransmissions may incur in additional undesired end-to-end delay, the authors propose correction mechanisms based on the transmission of redundant packets through multiple paths and the use of Forward Error Correction (FEC) codes, which are computed by each intermediate node instead of only in the source node and in the sink.

Source nodes encode the collected images following quality requirements specified by the sink. The images are coded using a wavelet-based image compression technique and a FEC Reed-Solomon code [36]. After transmission, multiple copies of the same packet may be generated in the network. Such redundant packets may be also combined and processed, resulting in new multiple copies of the same packet.

Employing the proposed solution, the errors are corrected as soon as possible, or the packet is discarded if the correction cannot be performed. In other words, hop-by-hop decoding and encoding is performed. Cluster heads perform recovery of entire packets that are lost during transmission (exploiting redundant packets) or of only bit errors in received packets (exploiting the FEC code). If only end-to-end correction was employed, early corrupted uncorrectable packets could be transmitted throughout the path, resulting in undesired energy wasting.

Error recovery in visual sensor networks is a key issue that may strongly impact the quality of the applications. Many works consider an error-free communication environment [33], which is unrealistic for most deployed wireless sensor networks, or they make artificial assumptions, such as constant loss rates [23]. The in-network error recovery approach investigated in [35] and the source approach proposed in [30] provide promising results for real-world sensor-based visual monitoring.

The image coding techniques aim to achieve efficient compression with error resilience, keeping an acceptable perceptual quality of the received media. In fact, there is a trade-off between processing and transmission tasks, where higher compression requires more computational resources and energy consumption. The work in [37] presents some execution and compression characteristics and discuss energy consumption in visual sensors.

We cannot forget that less-relevant information actually contributes to the final quality of the application. The fact that many works consider discarding of packets containing less-relevant information or reduction of the transmission rate of such packets may give a wrong feeling that such information is useless. Note that any data loss produces some degradation of the final image quality. What have been proposed are low and acceptable levels of degradation for an increased performance of the overall application in energy consumption, congestion control, error recovery and in-network compression. Table 1 summarizes the surveyed image-based cross-layer optimization solutions.

### 4.2. Video-Based Cross-Layer Optimization

Video transmission on visual sensor networks is even more challenging than the image-based transmission. In fact, video requires more bandwidth than image does, and the amount of data to be coded is much larger when source nodes are streaming video.

The coding process of collected video may follow different approaches, achieving high or low compression. For example, many techniques exploit information of the scene, such as motion and variation of luminosity, for higher compression. The expected resolution and number of frames per second (fps) are also relevant for the coding process. Whatever the chosen approach, the nature of resource-constrained wireless sensor networks will often bound the quality of the coded video, imposing low transmission rate and low resolution (typically CIF – 352 × 288 – or lower).

In video-based cross-layer optimization, protocols and algorithms in transport, network, MAC and even physical layers may exploit information of the coded data (application layer) to reduce the transmission rate, to decrease the end-to-end delay, to protect more relevant data against bit errors and packet dropping and to save energy in source nodes and throughout the network.

#### 4.2.1. Predictive Video Coding

Most research on video-based cross-layer optimization in the last few years has considered predictive encoding as the main option for video monitoring applications in resource-constrained wireless sensor networks. Among the possible reasons for such a tendency, predictive codecs have been widely used in the Internet, with many possibilities in compression levels and expected quality. Moreover, we have noted that the inner characteristics of such codecs are very propitious for cross-layer optimization, resulting in higher efficiency in visual sensor networks.

The work in [38] regards multipath predictive-encoded video transmission in wireless sensor networks, proposing two packet scheduling algorithms to be used when the aggregated bandwidth of the paths is lower than the transmission requirements of the applications. The idea is to discard less relevant frames in order to decrease the source transmission rate, adapting to the current resources of the sensor network.

The authors define a recursive distortion prediction model to identify the expected relevance of the frames. The proposed prediction model regards different types of bit errors during transmission (isolated, burst of losses and errors separated with a small lag) [39,40], since the nature of the error can influence the quality of the received media. Based on this model, the proposed algorithms know what frames could be discarded prior the transmission, without increasing significantly the video distortion in the receiving side.

In that proposal, the images collected by sensor nodes are encoded using the H.264 codec, due to its high compression efficiency. This codec uses previously encoded frames as reference for motion-compensated prediction. In experiments, the authors consider a frame rate of 30 fps with QCIF resolution (176 × 144), and encoded data being transmitted in RTP (Real-time Transport Protocol) [41] packets of 1,024 bytes size.

The proposed algorithms in [38] operate on the cluster-based routing protocol LEACH (Low-Energy Adaptive Clustering Hierarchy) [42], which was adapted to support multipath routing and improve the energy consumption on cluster heads. Despite that, the original data is only processed by the source node, in a different way from [33–35]. The proposed recursive distortion prediction model is very promising for visual sensor networks. That model considers a robust error theory which can be useful for real-world implementations. Some drawbacks rely on the resulting complexity for constrained-resources sensors and the use of UDP/IP mechanisms. Internet-based transport and network protocols are unsuitable for most wireless multimedia sensor networks, as already stated by the academic community [5,6].

Following this same investigation line, the work in [43] proposes a video compression logical sub-layer which defines a new compression model able to prioritize frames. Moreover, the authors define protocols for the transport and network layers. The proposed solution is defined as the Energy-efficient and high-Quality Video transmission architecture (EQV-Architecture). In short, the EQV-Architecture extends the lifetime of the network assuring the sufficient level of the video quality.

The proposed compression model defines the M-MPEG codec (Modified-MPEG) based on MPEG-2. Compression is accomplished through M-frames and D-frames, all created using an extension of JPEG to prioritize image blocks. The main idea of this work is to create more relevant encoded frames that should be transmitted through highly reliable schemes, while flows of less relevant frames use semi-reliable schemes. Among the proposed solutions, more relevant frames are subject to Reed-Solomon forward correction codes.

The type of the employed transmission service (reliable or semi-reliable) is structured over two packet dropping strategies: energy aware dropping and random early dropping. In the first one, the priority levels for packet dropping are calculated regarding the normalized energy levels of the intermediate nodes, and each node has a particular priority level. In such an approach, all received packets with priority level equal or lower than the current node priority are discarded. For this approach, the energy consumption over the network is considered, avoiding dropping packets that are close to the sink (since they have already consumed energy of previous intermediate nodes). In the second dropping scheme, less relevant packets have a probability to be early dropped (before transmission), avoiding undesired energy consumption with low prejudice to the end-to-end quality of the application. Such a dropping strategy resembles the proposed scheduler algorithm of [38], but with slight different objectives.

Considering the whole proposed architecture in [43], it is expected that the user can adjust the energy consumption thresholds and the desired video quality and bandwidth usage directly in the proposed compression sub-layer. Video resolution varies according to the application requirements and it is also adjustable, but the received video is always in gray-scale.

Cross-layer optimization based on predictive encoding is also exploited in Chen *et al.* [44]. Multipath routing is used to transmit encoded video using H.26L coding technique, where a path priority scheduling algorithm is employed. The paths computation is based on Directed Geographical Routing (DGR) protocol [45], which creates multiples node-disjoint paths.

In order to maintain high application quality, the paths with lower delay should be used more often to transmit packets containing encoded video. The idea is to exploit information about the available bandwidth, the expected end-to-end delay and the residual energy of the paths, assigning video substreams to the “better” paths. The schedule algorithm aims to protect critical paths and balance traffic load among the available paths, where only paths which meet the delay requirement of the application may be selected. For the algorithm, higher energy, higher bandwidth and higher delay paths will be scheduled more frequently, protecting the lower delay paths and still assuring that the maximum delay tolerated by the application is respected. Moreover, packets containing more relevant data are assigned to paths with lower delay, higher bandwidth and larger energy, since a path with less capacity and energy will more likely fail during the transmission.

Other key service of the proposed solution is early selective packet dropping, similarly to [38]. If the required bandwidth is larger than the aggregate bandwidth of the available paths, packets with the least importance will be discarded, resulting in a potential small degradation of the perceived quality of the decoded video.

Predictive encoded video can also be processed while transmitted through the sensor network. As video encoding is computing-intensive and requires low delay and jitter, when compared with images, most works regard in-network processing for energy preservation and error recovery.

The work presented in [44] exploits in-network processing to achieve multimedia-based cross-layer optimization. When an intermediate sensor node receives a packet to be relayed, it estimates the packet delay to the sink. If the estimated delay is larger than the required delay by a certain threshold, it assumes the packet is highly likely to miss the frame deadline, and so it discards the packet. Additionally, the intermediate node sends an ISLER (Inform Source to Lower Encoding Rate) message to the source node. Upon reception of this message, the source node updates information about the path and the video coding (based on H.26L) is adjusted.

The work presented in [46] proposes multimedia-based cross-layer error recovery, where predictive encoding is performed at source nodes. In the proposed scheme, the most relevant frames for the decoding process are transmitted through paths with lower loss probability, where the loss probability is measured from the network. If such packets are lost, the receiver end may request retransmission. But as end-to-end retransmission is performed, it can incur in excessive delay and should not be considered for real-world VSNS applications [47,48].

Multimedia-based cross-layer optimization can also consider MAC protocols. Zhang and Ding [49] propose differentiated treating of multimedia packets by MAC layer according to the relevance of the encoded data in wireless multimedia sensor networks. The predictive video codec MPEG-4 is exploited as well as the IEEE 802.11s MAC protocol. MPEG-4 codec defines three types of video frames: I-frame, P-frame and B-frame. The most relevant data for the decoding processing are I-frames, followed by P-frames and B-frames.

The 802.11s MAC protocol defines four classes of traffic (AC3 for voice, AC2 for video, AC1 for best effort and AC0 for background traffic), each one directly related to the priority to access the communication medium. As already expected, video streaming is transmitted through the AC2 traffic class.

Because intermediate nodes that compose an active path from one source to the sink may be equipped with a CMOS camera or a scalar sensing unit, these intermediate nodes may be relaying packets from other sources and transmitting packets produced by themselves. An important issue addressed by [49] is the differentiation of forward and local packets: As forward packets have already traversed several hops, they should be prioritized against local packets (produced by the intermediate node itself). The communication scenario where intermediate nodes may forward and produce packets is also investigated in [50]. In that work energy saving is achieved reducing the traffic load on paths that are comprised of intermediate nodes which are able to produce relevant visual information for the monitoring application.

There are two major contributions in [49]. In first place, the AC2 queue may overflow if many video packets are being received, resulting in packet dropping. Such congestion is addressed by a selective algorithm that drops packets based on the relevance of the encoded data according to the MPEG-4 coding technique, reducing the impact on the perceptual quality of the received video. The second proposed solution tries to avoid congestion by performing queue management in advance. If the current size of AC2 queue is higher than a threshold, incoming (forward) packets are allocated to AC1 queue, while locally produced packets are assigned to AC0 queue. Such scheme assures that forward packets have higher transmission priority than local packets.

The work in [49] proposes a multimedia-based cross-layer regarding the IEEE 802.11s MAC protocol. In fact, this MAC protocol is mainly intended for mesh networks, where energy is not a major concern as in WMSNs. Although authors indicate the proposed solution for wireless multimedia sensor networks, further investigation should be performed to assess the feasibility of IEEE 802.11s in energy-constrained low-cost wireless sensor networks.

Other works in the literature investigate multimedia-based cross-layer optimization focusing MAC protocol IEEE 802.11e, as [51] for H.264 and [52] for MPEG-4. However, neither of these works considers energy as a key design issue.

Algorithms to prioritize packets according to their relevance for the decoding process [38,43,44,46,49] are very promising for VSN applications, achieving relevant results in end-to-end delay, perceived visual quality and energy consumption. This is significantly different from control of the source transmission rate not regarding the inner relevance of the coded data, as in [53]. In fact, it is expected higher overall efficiency in selective multimedia-based packet dropping, error recovery and congestion control mechanisms when compared with traditional multimedia-unaware solutions.

#### 4.2.2. Multiple Description Coding

Multiple description encoding [54,55] may potentially benefit wireless multimedia sensor networks. In recent years, such techniques have been exploited for multipath routing, especially when sensor nodes collect visual and audio data from the monitored field.

The context-aware multimedia-based cross-layer optimization scheme proposed in [12] exploits multipath routing along with the relevance of the encoded data for efficient path selection regarding the end-to-end communication delay. It is not properly specified a coding technique, but the proposed routing scheme is very suitable for multiple description coding.

That work defines the MPMPS (Multi-Priority Multi-Path Selection) algorithm to find the paths with lower end-to-end delay for multimedia streaming in WMSNs, considering a set of available node-disjoint paths. Such paths are node-disjoint when they have no common intermediate nodes. The node-disjoint paths are discovered employing the Two-phase Geographic Greedy Forwarding (TPGF) algorithm [56], which finds the maximum number of optimal node-disjoint routing paths in terms of path length and the end-to-end transmission delay, potentially benefiting wireless multimedia sensor networks applications. The authors argue that traditional multipath routing protocols do not provide a powerful searching mechanism to find the multiple optimized routing paths and to bypassing holes [56,57].

**Figure 2.**
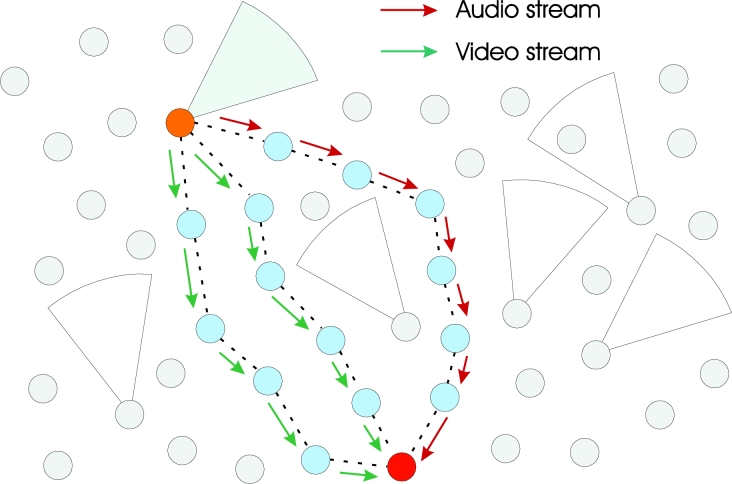
Multipath multimedia transmission through three node-disjoint paths.

Figure 2 shows a visual example of multiple node-disjoint paths from one source to the sink. Note that video data are split in two streams that flow over potentially lower-delay paths (four intermediate nodes). In that example, the unique audio stream flows over a path comprised of six intermediate nodes.

The work presented in [12] proposes the splitting of the source stream into image and audio substreams, giving to each resulting substream a particular priority according to the current monitoring being performed. The paths with lower delay are assigned to the higher priority substreams, leaving the remaining paths to the lower priority substreams. The expected delay is measured by the number of intermediate nodes of the paths (the less is the number of intermediate nodes, lower end-to-end delay is achieved).

The authors of [12] cite an interesting communication scenario for the proposed solution. In fire monitoring, visual information is more relevant for the application and should be delivered with minimum transmission delay. The audio stream could be transmitted over the remaining paths, complementing the received visual data. In practice, as some available paths may have higher transmission delays than the time constraint of the application, they are not considered by traditional single description coding applications (audio and image together). On the other hand, even available paths with high delays could be used by the application for transmission of the lower-priority substream in [12], maximizing the attainable communication throughput.

The concept of context-aware multimedia-based cross-layer optimization presented in [12] is further investigated in [57]. In both works, the original data (72 kbps) is split into an image stream (48 kbps) and an audio stream (24 kbps). A generic treatment of multistream multipath transmission is given in [58].

Li *et al.* [59] exploit multiple descriptions coding for multipath-selection. However, while TPGF was employed in [12,57] to create multiple node-disjoint paths, the work in [59] extended the Direct Diffusion protocol [60] to discover multiple node-disjoint paths. This is in fact the main contribution of [59], since MDC is employed just to assess the performance of the proposed multipath routing protocol.

The work presented in [46] also combines MDC with multipath transport. However, in a different way from [12], the proposed scheme continuously monitors the path to decide the number of substreams that have to be created. Among the three video transport techniques proposed in [46], only one is suitable for WMSN, since it does not required feedback messages to be sent for each frame by the receiver end, which could increase the energy consumption and the end-to-end delay. Nevertheless, as the energy consumption for any of the proposed schemes was not evaluated in this work, their using in visual sensor networks may be not feasible, although their contributions have influenced other works.

#### 4.2.3. Distributed Video Coding

Predictive coding is dominated by motion estimation, with the complexity relying on the encoder side. According to Girod *et al.* [61], predictive video encoders may be indeed 5–10 times more complex than decoders. For visual sensor networks, such complexity may demand more computational power of source nodes for the encoding process, also resulting in additional energy consumption. A feasible solution to shift the complexity to the decoder side is to employ DVC as the coding technique.

Distributed video coding is a video compression paradigm based on Slepian and Wolf’s [15] and Wyner and Ziv’s [16] theoretical results. The idea is to transmit intra-coded frame along with side information frame. The intra-coded frames are generated using some technique for image processing, as DCT or pixel-based compression [62]. The side information frames are intended to be processed at the decoder, which exploit the temporal correlation among the frames. As motion information is only considered at the decoder side, DVC demands less computational power and energy at the encoder side than predictive video coding, also providing more error-resilience [63].

Most works in the literature that consider DVC as the coding technique are concerned with quality distortion due to bit errors and packet dropping. In this way, Xue *et al.* [64] defined a communication environment comprised of a main camera sensor with high processing capabilities and many deployed wireless sensors to cover different views of the target scene. The idea is to code the visual data from the main camera using usual intra-frame coding techniques, encoding the remaining data from the other sensors with DVC. The DVC scheme Pixel-Domain Wyner-Ziv (PDWZ) is employed along with an adaptive rate control mechanism intended to control the number of parity bits based on feedback from the decoder side. The transmission adjustment in that work is based on the application layer, subject to feedback messages transmitted from the decoder side.

Liang *et al.* [65] define an unequal error protection scheme to assign different protection levels to the different elements of a DVC compressed video. In the feedback aided unequal error protection scheme, a feedback channel provides information that are used to adjustment the parity data rate. A second scheme adjusts the correction data according to the amount of motion information being transmitted by the source.

The work presented in [66] proposes a rate control method that considers the channel loss and the correlation of video images, providing more resilience to packet errors when transmitting DVC encoded video. The rate adjustment is performed controlling the quantization parameters of the DVC encoding, adjusting the number of parity bits for error-resilience. Doing so, the expected overall quality for the proposed method is higher than the conventional method (adjustment based only in correlation of video images) when transmitting packets over error-prone links.

Both works presented in [65] and [66] adjust the amount of parity data according to the current state of the network and/or the relevance of the transmitted coded data. Despite the fact that their results contribute to error recovery in VSNs where DVC is employed, neither of them conducts sufficient analysis on the energy consumption in source nodes throughout the active paths.

Distributed video coding is being constantly proposed as the most suitable coding option for wireless multimedia sensor networks, but we are not convinced of that. It is a fact that DVC achieves energy saving in source nodes, shifting the complexity to the receiver end, but most energy consumption is expected to occur in the communication process, and predictive coding achieves high compression. Other relevant consideration is that for many applications, monitoring is not expected to be performed until the complete energy consumption of the nodes. For example, Shu *et al.* [67] defined a monitoring environment where energy saving is not a major issue, but end-to-end delays have to kept in a low level. Finally, many works that have proposed predictive video-based cross-layer optimization have provided some feasible ways to balance the drawbacks of such video coding techniques. We are not advocating that predictive coding is more suitable than DVC. What we believe is that there is no preferred multimedia coding technique (for image and video alike) in visual sensor networks, since the application requirements and the characteristics of the deployed network will strongly influence the choosing of the proper codec.

It is interesting to note that the end-to-end delay is a major concern of video-based wireless sensor networks applications. Multipath routing, hop-by-hop loss recovery [6,48] and transmission of redundant packets may increase the quality of the received media with low impact to the end-to-end delay. As an example, the works presented in [68,59] considers that the maximum delay should be lower than 200 ms. Additionally, the maximum delay for [12,57] is 280 ms. Table 2 summarizes the surveyed video-based cross-layer optimization solutions.

## 5. Research Directions

In the last few years, many cross-layer optimization approaches exploiting characteristics of multimedia coding have been proposed. Such works present a wealth of contributions to energy preservation, transmission rate adjustment, congestion control, error recovery and multipath selection. However, the problem of multimedia streaming in resource-constrained visual sensor networks is not yet completely solved, and new challenges are still arising. We discuss some research directions for cross-layer optimization based on multimedia coding.

A natural consideration to guide future research is the way multimedia coding techniques might evolve. When analyzing the last twenty years we note a clear evolution line of audio, image and video coding techniques. In visual sensor networks, new codecs have been considered all the time, with different results in computational complexity, data compression and error resilience. Old standards have been further enhanced, while new approaches as distributed video coding and in-networking processing have been considered for visual sensor networks.

Regarding the evolution of multimedia coding, it is worth wondering about what information is actually relevant for a given application. For example, the work in [69] investigates video compression using address-event representation, which intends to achieve frame-difference coding with low computational cost. Frame-difference data can be used to indicate motion of a moving target in VSNs. The authors argue that for some applications, information about the moving behavior of a target is more relevant than the visual information itself. Doing so, the identity of the target may be preserved, but the observer can still be able to understand the actions of the target. We must also outline that too few bytes are necessary to present frame-difference information, when compared even with grayscale images.

The evolution of the coding approach can be even deeper. Pudlewski and Melodia [70] conducted a cross-layer performance evaluation of Compressed Sensing video streaming in WMSNs. Compressed sensing is a new paradigm that claims to allow the faithful recovery of information requiring fewer measurements than traditional sensing. It is shown that CS-encoded images exhibit an inherent resiliency to link errors, unlike JPEG images, due to unstructured image representation.

Based on the works surveyed in the previous sections, we can expect the evolution of the coding and sensing paradigms will influence the cross-layer optimization in VSNs. But there are other issues that should be properly considered. The work in [13] defines a tracking system for wireless visual sensor networks, where there are multiple mobile sinks. This potentially changes the way protocols perform path selection, congestion control and error recovery, as well as the coding strategies for multimedia streaming. Campelli *et al.* [68] consider sensors endowed with an ultrawideband (UWB) transceiver. Although UWB may considerably increase the sensors costs, this physical layer technology allows transmission rates much higher than the ZigBee technology [2]. As UWB is claimed as the ideal physical layer technology for wireless multimedia sensor networks [5,6], cross-layer multimedia coding will have to consider the benefits and streaming challenges of *ad hoc* sensor networks where intermediate nodes are interconnected by UWB-enabled links.

The adopted MAC protocols may also influence the multimedia-based cross-layer design. For example, IEEE 802.11 technology is considered as the link-layer technology for [20,38,46,59] and IEEE 802.15.4 is used in [19]. We expect that those MAC protocols will be not employed in near future real-world VSNS applications, mainly due to energy constraints of the sensor nodes. However, some energy-aware MAC protocols have been considered for wireless multimedia sensor networks, as T-MAC [71] in [30] and ET-MAC [72] in [43].

The nature of the wireless links may also impact the cross-layer architectures, but in a different way. In general, packet dropping in visual sensor networks is a result of network congestion or bit errors. When packets are transmitted through wireless links, there is a probability that bit errors occur during the transmission. Many works expect a linear error probability over a single bit, but such consideration is unrealistic. In fact, bit errors appear in bursts and large packets are more likely to be discarded than small packets [39,40]. As smaller packets lead to additional protocol header overhead, researchers should be worried about the ideal size of packets carrying multimedia encoded data. An interesting approach is cross-layer packet size optimization, as discussed in [40]. However, most of the survey works have no clear considerations of the size of transmitted packets. When the packet size is defined, as in [38], which defines RTP packets of 1024 bytes, a clear arguing of the reasons for such choice is missing.

As energy constraints should remain a major concern for the upcoming years, multimedia-based cross-layer design in visual sensor networks has to save energy while achieving the desired optimization, or at least not incur in additional energy consumption. There are many energy-unaware works in the literature proposing multimedia-based cross-layer optimization [73–75]. Future research could further enhance their contributions adding energy saving mechanisms, achieving feasible solutions for visual sensor networks.

New challenges in transmission rate adjustment, energy preservation, congestion control, error recovery, multipath transmission and in-network compression should still arise, requiring additional research in multimedia-based cross-layer optimization. Some of these challenges may be more stringent in specific monitoring applications, concerning for example multi-tier architectures [76,77] or heterogeneous sources as described in [64]. Other issues as source nodes mobility and coverage preservation also request from the academic community new research efforts, directly impacting the employed coding technique and the adopted cross-layer solution. Finally, priority-oriented scheduling based on appropriate computer vision techniques, such as foreground distinguishing, should also be considered in future work [78,79].

## 6. Conclusions

Multimedia-based cross-layer optimization exploits the inner characteristics of multimedia coding techniques together with the joint design of network protocols to achieve higher efficiency in visual sensor networks. We have surveyed the state of the art of research addressing such particular issues, presenting relevant contributions in the fields of transmission rate adjustment, energy preservation, congestion control, error recovery, multipath selection and in-network compression. The drawbacks of the surveyed works were also discussed. Finally, future research directions were presented, indicating promising investigation areas regarding this issue. We believe that multimedia-based cross-layer optimization will become dominant in modern visual sensor networks.

## Figures and Tables

**Table 1.** Image-based cross-layer optimization.

**Approach**	**Coding**	**Processing**	**Optimization**
Boukerche *et al.* [20]	Progressive	Source	Transmission rate adjustment.
Cheng and Shang [22]	Progressive	Source	Transmission rate adjustment.
Leelapornchai and Stockhammer [23]	Progressive	Source	Multipath routing. Error recovery by correction codes.
Lecuire *et al.* [24]	Wavelet-based	Source	Congestion control. Energy preservation.
Lee and Jun [26]	Wavelet-based	Source	Congestion control.
Yu *et al.* [19]	Wavelet-based	Source	Transmission rate adjustment.
Wang *et al.* [29]	Any	Source	Energy preservation.
Wang *et al.* [30]	Wavelet-based	Source	Error recovery by correction codes.
Wu and Abouzeid [33]	Wavelet-based	In-network	In-network multimedia compression.
Nasri *et al* [34]	Wavelet-based	In-network	In-network multimedia compression. Transmission rate adjustment.
Wu and Abouzeid [35]	Wavelet-based	In-network	Redundancy-based error recovery. Error recovery by correction codes.

**Table 2.** Video-based cross-layer optimization.

**Approach**	**Coding**	**Processing**	**Optimization**
Politis *et al.* [38]	Predictive	Source	Transmission rate adjustment.
Aghdasi *et al.* [43]	Predictive	Source	Transmission rate adjustment. Energy preservation. Error recovery by correction codes.
Chen *et al.* [44]	Predictive	Source/In-network	Transmission rate adjustment. Multipath routing. Energy preservation.
Mao *et al.* [46]	Predictive	Source	Multipath routing.
Zhang and Ding [49]	Predictive	In-network	Differentiated MAC transmission.
Zhang *et al.* [12]	MDC/Any	Source	Multipath routing.
Shu *et al.* [57]	MDC/Any	Source	Multipath routing.
Li *et al.* [59]	MDC	Source	Multipath routing.
Mao *et al.* [46]	MDC	Source	Multipath routing.
Liang *et al.* [65]	DVC	Source	Error recovery by correction codes.
Kim *et al.* [66]	DVC	Source	Error recovery by correction codes.
